# Supplementary material for: Identification of photoperiod-induced specific miRNAs in the adrenal glands of Sunite sheep (Ovis aries)
Source: Front Vet Sci. 2022 Jul 22;9:888207. doi: 10.3389/fvets.2022.888207 (PMC9354845; doi:10.3389/fvets.2022.888207)
Supplement: Supplementary file 1 [file Data_Sheet_1.ZIP › Supplementary file/miRDeep2 file.zip!/miRDeep2 file/Novel_Structure/SPLP42A1/Novel_703.pdf]

| | | |
|---|---|---|
| Provisional ID | : | NC_019484.2_31773 |
| Score total | : | 17.8 |
| Score for star read(s) | : | 3.9 |
| Score for read counts | : | 10.3 |
| Score for mfe | : | 2 |
| Score for randfold | : | 1.6 |
| Score for cons. seed | : | |
| Total read count | : | 32 |
| Mature read count | : | 23 |
| Loop read count | : | 0 |
| Star read count | : | 9 |

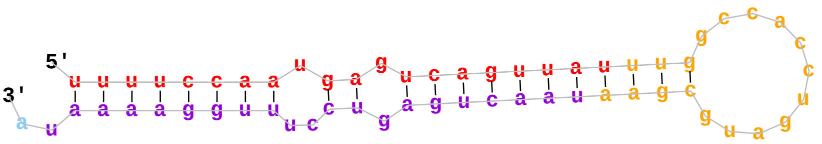


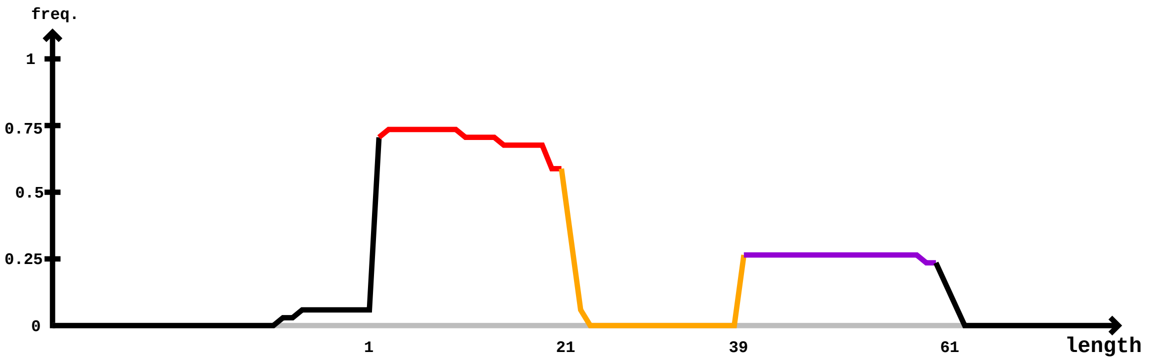


Mature Star

```
5'- ucucccuguuuuaaaacuucaacaucagggucuuuuccaaugagucaguuauuuggccaccugaugcgaauaacugaguccuuuggaaaauacccugaugcugggaaag  -3'  obs
    ucucccuguuuuaaaacuucaacaucagggucuuuuccaaugagucaguuauuuggccaccugaugcgaauaacugaguccuuuggaaaauacccugaugcugggaaag        exp
    ..((((.(((....))).....(((((((((.((((((((.((.(((((((((((...........))))))))))).))..)))))))).)))))))))..))))...  reads  mm  sample
    ......................caucagggucuuuucAaau....................................................................  1  1  seq
    ........................ucGgggucuuuuccaaugagu................................................................  1  1  seq
    ................................uuuuccaaugagucaguu...........................................................  3  0  seq
    ................................uuuuccaaugagucaguuau.........................................................  8  0  seq
    ................................uuuuccaaugagucaguuaGu........................................................  1  1  seq
    ................................uuuuccaaugagucaguuUuu........................................................  1  1  seq
    ................................uuuuccaaugGgucaguuauu........................................................  1  1  seq
    ................................uuuuAcaaugagucaguuauu........................................................  2  1  seq
    ................................uuuuccaCugagucaguuauu........................................................  1  1  seq
    ................................uuuuGcaaugagucaguuauu........................................................  1  1  seq
    ................................uuuCccaaugagucaguuauu........................................................  1  1  seq
    ................................uuuuccaaugagucaUuuauu........................................................  1  1  seq
    ................................uuuuccaaugagucaguuauuA.......................................................  2  1  seq
    .................................uuuccaaugagucaguuauu........................................................  1  0  seq
    ......................................................................uaacugaguccuuuggaaa....................  1  0  seq
    ......................................................................uaacugaguccuuuggaaaau..................  6  0  seq
    ......................................................................uaacugaguccuuuggaaaaua.................  2  0  seq
```
